# Insect visuomotor delay adjustments in group flight support swarm cohesion

**DOI:** 10.1038/s41598-023-32675-5

**Published:** 2023-04-19

**Authors:** Md. Saiful Islam, Imraan A. Faruque

**Affiliations:** Oklahoma State University, Stillwater, OK USA

**Keywords:** Aerospace engineering, Systems analysis

## Abstract

Flying insects routinely demonstrate coordinated flight in crowded assemblies despite strict communication and processing constraints. This study experimentally records multiple flying insects tracking a moving visual stimulus. System identification techniques are used to robustly identify the tracking dynamics, including a visuomotor delay. The population delay distributions are quantified for solo and group behaviors. An interconnected visual swarm model incorporating heterogeneous delays is developed, and bifurcation analysis and swarm simulation are applied to assess swarm stability under the delays. The experiment recorded 450 insect trajectories and quantified visual tracking delay variation. Solitary tasks showed a 30ms average delay and standard deviation of 50ms, while group behaviors show a 15ms average and 8ms standard deviation. Analysis and simulation indicate that the delay adjustments during group flight support swarm formation and center stability, and are robust to measurement noise. These results quantify the role of visuomotor delay heterogeneity in flying insects and their role in supporting swarm cohesion through implicit communication.

## Introduction

A significant barrier to creating adaptive swarms of small scale unmanned aerial systems and other challenging robotic swarms remains the provision of fast, computationally-lightweight sensing and feedback structures to support relative navigation in dynamic groups^1–3^. Insects flying in groups can serve as model systems for resource-constrained feedback on these swarming micro air vehicles^4^. Despite tight constraints on sensory and neural feedback mechanisms and the lack of a conventional communication network, insects in naturalistic swarms coordinate flying movements in close proximity to dynamically changing numbers of neighbors in unstructured environments. Visual control may be a critical tool for implicit communication, given the large fraction of insect neural resources dedicated to visual processing. Vision is one of the few insect sensor modalities with quantifiable bandwidth, range, and sensitivity that could provide real-time data to adjust trajectories^5,6^, yet the specific processes supporting inflight feedback in insect group behaviors remain unknown. Many theoretical swarm models and numerical studies have not yet been integrated with experimental investigations on naturalistic swarms, limiting the ability of theory and experiment to inform each other. Group behavior implies that complex interactions may be used to make individual and aggregate decisions^7–9^. Direct application of stimuli to swarm can result in deviations from their usual biological to adaptive behaviors^10–12^. The effects of environmental stimuli examining insect flight behavior and motions during visually-dominated behaviors like obstacle avoidance, landing on a wall or proboscis, and flower tracking have previously focused most on the role of ambient and external illumination levels^13–15^. Insect flight trajectories can be tracked and recorded through several existing software tools^16–20^. For studies involving precise timing of an insect or collection of insects tracking a visual stimulus, the recording of visual stimulus in the background is also necessary for accurate analysis. Midge swarms regulate themselves relative to a dynamic moving stimulus which reveals possible interactions present in their common activity^21^. Swarm markers and light intensity experiments with *Chironomus riparius* in a laboratory environment indicated that pheromones can be a important role in swarm cohesion, and these midge swarms suggest that relatively small numbers of agents (e.g., 10 agents) are sufficient to saturate statistical measures of swarm behavior^22,23^. When flying in wind, the unsteady flight of a hawkmoth in the wake of a 3D printed robotic flower displays larger tracking overshoot and a reduced order dynamic system^24^ and a flower tracking experiment was used to quantify the change in their flight behavior under various light conditions, with average flower tracking behavior represented by a simple temporal delay at various light intensities^25^. By adjusting light intensity, a system identification approach was utilized to find a brightness-dependent delay term in the transfer function, resulting in a dynamic model for each Hawkmoth variant that included a combination of species-dependent scaling parameters and processing delays^26^. Neuronal networks with delays in biological examples can have a significant role on observed biological behaviors, including generating oscillatory motions and periodic signals^27–29^. Despite the rich history of experimental study, measurements of how processing delays are distributed among swarming individuals are not yet available.

**Figure 1:**
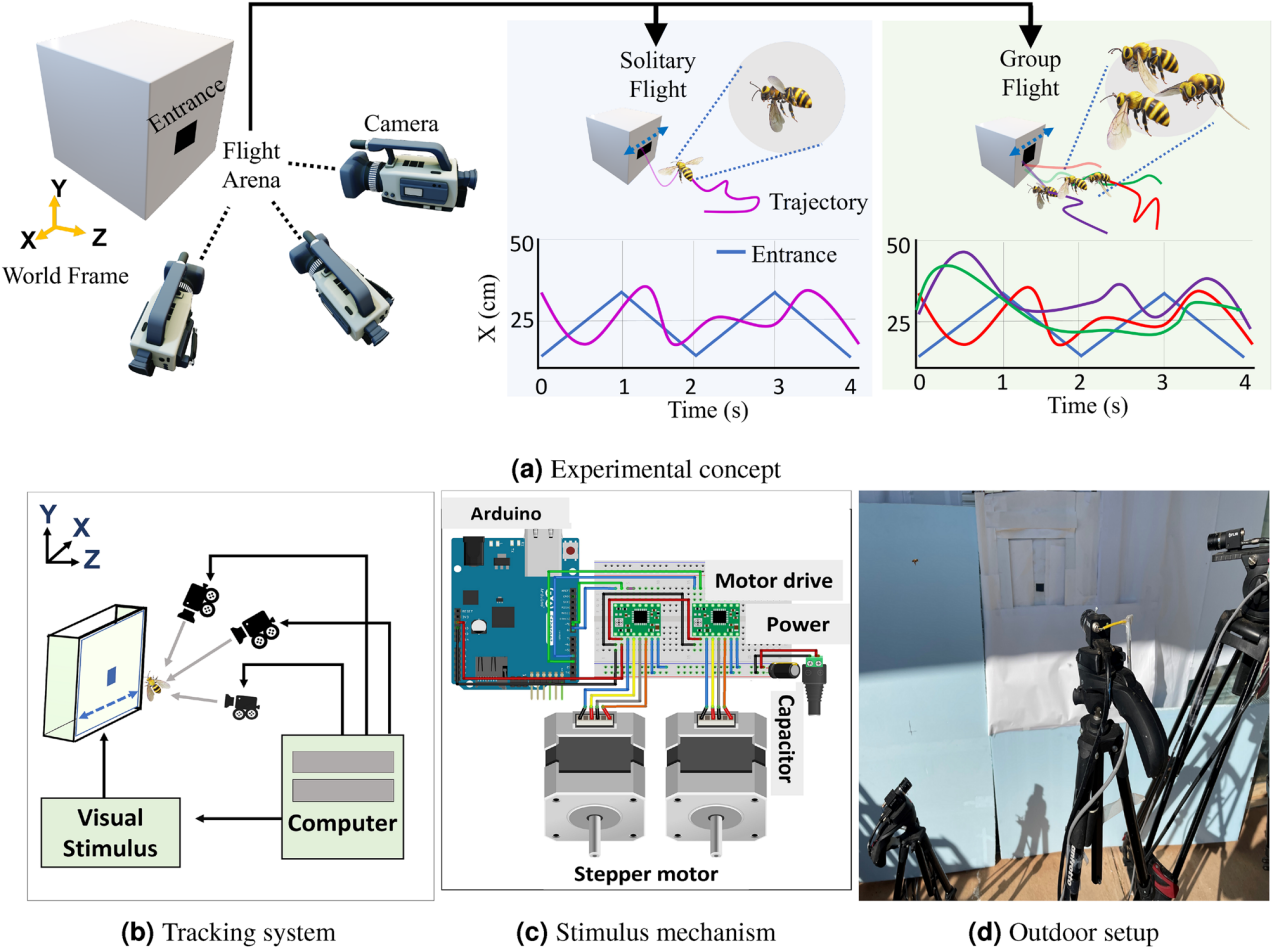
(**a**) Experimental design consists of three camera based tracking system which record the trajectories of insects approaching to the entrance. Two types of bees’ data were segmented: solitary flight (light blue shading) and group flight (light green shading). X coordinates of entrance (blue) and insect from their 3D trajectories are considered as input and output for system identification approach, respectively. A three camera-based tracking system (**b**) was used to record flight paths of multiple insects (and stimulus) tracking a moving hive entrance actuated by an Arduino micro-controller and stepper motors (**c**), mounted in an outdoor environment (**d**).

Mathematical descriptions of collective motions of multi-agent biological systems, including bacterial colonies, slime molds, locusts, and fish, have become available in the last few decades^30–32^. These models range from continuous approximations and kinetic theory structures to models at the individual level^33–36^, with interaction mechanisms often consisting of attractive and repulsive forces^37,38^ affecting particle motion. Swarm characteristics such as cohesion, velocity alignment, and predator avoidance can be achieved in swarms of dynamic models via bifurcation parameters located in the attractive and repulsive potentials^39–41^. An individual agent’s influence on swarms is posed using zone based models and communication topological analysis indicates that the attraction and alignment weights affects the group behavior and structure^42^. The swarm’s collective behavior can also be described by elastic or thermodynamic analogies where external stimulus can represent the midge swarm behavior^43^. Despite the availability of rigorous proofs for sufficiency of velocity alignment (e.g.^44^), theoretical models have often relied on a high level of instantaneous connectivity that may be impractical in nature or robotic implementations. While experimental research is beginning to understand the need to quantify internal delays due to insect sensing and feedback, these previous studies are limited to reporting a single average delay across all animals and do not yet account for the heterogeneity of delays across the population or the effect of such delays on neighbor-coordinated behaviors. Delay differential equations (DDE) have been used to explore biological delay models with homogenous delays or delays belonging to discrete or continuous distributions^45–48^. Recently, rigorous mathematical analysis and role of delays such as auto regulations, feedback loops, etc. in biological models have been used to describe the quantitative and qualitative behavior of such systems analytically^49^. Lyapunov analysis has recently been used for time delays on swarms composed of first and second order systems to find conditions for swarm stability under homogeneous delays^50^. The theoretical effects of delay in such a network of interaction rules has previously been investigated primarily numerically by distributing delays among agents and applying a mean field analysis^51,52^. The delays across the agents were modeled as heterogeneous, drawn from a Gaussian distribution.

A critical need is understanding the way in which these animals manage individual sensing and feedback processing delays, which may be dynamic or show population-wide diversity. Many animals (and aerial robotic implementations) show a nonzero latency or a reaction delay due to sensory processing. The effect of individual agent latency heterogeneity in swarming experiments has not been adequately related to theoretical findings, with experimental studies reporting average delays rather than quantifying the distributions of individual delays. We previously quantified the visual reaction time seen in flying insects tracking a moving light in a solitary task^53^. This laboratory experiment found that honeybees’ reaction times were diverse, varying from 4 to 115ms. The identified reaction times were measured at the individual insect level, and probability distributions were experimentally quantified for the measured delays. We used theoretical swarm communications analysis and simulations of a swarm responding to neighboring animals including these delays, which indicated that the swarm level effect of the varying reaction time is to damage the cohesion of swarm motions. This experiment was conducted in a laboratory environment using captured bees, which might influence them not to perform their normal behavior. Would a more naturalistic outdoor experiment give the same reaction time variation?

To test the **Hypothesis (H1)** that *the distribution of insect in-flight reaction times may be different outdoors*, we designed a more naturalistic outdoor flight experiment to record visual tracking trajectories outdoors as seen in Fig. 1. The tool divides into two parts: visual stimulus (Fig. 1c) and tracking system (Fig. 1b). Horizontal stimulus movement design is covered in section “Stimulus design”. This experiment expanded to multiple insect tracking (section “Imaging system”). With three or more cameras placed in front of the experimental setup, VISIONS system can measure multiple insects’ 3D positions at 60–120 Hz. We segmented insects’ trajectories while they approached and entered the moving entrance. We identified systems in both the time and frequency domains between the moving stimulus and the insect position. Frequency domain identification is accomplished when record lengths are sufficient (>1.5 s) and time domain when records are shorter. The identified dynamics are then compared between solitary and group flight. This system identification methodology also performed well under noisy data. The experimental results indicate that the insects have heterogeneous processing delays, and both the heterogeneity and magnitude of the delay are reduced in group tasks, with two different gamma distributions fitting the data. Experimental work on delay quantification and theoretical work on delay modeling have largely remained distinct, and there is a need to understand how measured delays affect swarm behavior. To fulfill the need, an attractive and repulsive potential-based swarm model incorporating processing delays was introduced. Two weights (egocentric and neighbor influence) and a processing delay parameterize individuals in the visual swarm. The model integrates the measured delay distributions to predict regions of stable and unstable swarm behaviors by mean field and bifurcation analysis, and a simulation of the modeling framework verifies that the swarm behavior stability results persist with experimental delays and for relative position stability.

The main contribution of this paper is to use experimental and theoretical tools to investigate the influence of visual delays on insect swarm behavior. This study connects these areas to address several questions: (a) what is the population-wide distribution of insect in flight processing delays, (b) does this distribution change in solo vs group behaviors, and (c) if so, how does it affect visually-guided multi-agent (e.g., group and swarming) behaviors?

## Results

We analysed 450 trajectories, segmented into 225 solitary insect trajectories and 225 group behavior trajectories. As seen in Table 1, a similar percentage of solo and group trajectories met length requirements for frequency domain identification (51% solo vs 48% group), while the remainder (49% and 52% of trials) were completed in time domain. Average fit percentages remained above 80% with standard deviations from 8-16%.

**Table 1:** Overall identification statistics.

Role	Frequency domain			Time domain		
	Trials	Avg.	Std.	Trials	Avg.	Std.
Solo	115	84%	13%	110	79%	12%
Group	106	80%	11%	119	81%	8%

Before discussing overall properties of the identified models, examples of individual identifications in each category are shown.

### Solitary dynamics identification

We conducted individual dynamics identification by adapting our previous experimental work, which incorporates both time and frequency domain approaches. The strength of these results rely on an ability to inject input stimulus that are tailored to excite the internal dynamics, and input stimulus design was a major methods consideration.

#### Example: frequency domain identification

**Figure 2:**
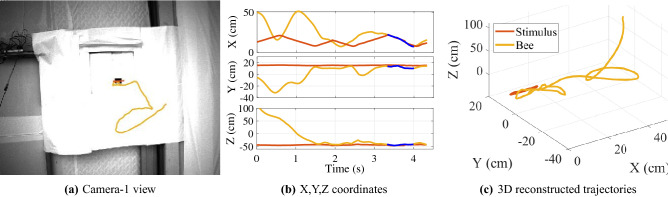
(**a**) A solitary insect’s trajectory (length 4 s) entering the moving entrance is captured by camera-1; (**b**,**c**): reconstructed position coordinates and 3D trajectory for both input stimulus and insect (blue color indicates Kalman filter).

When length criteria were met, frequency domain identification was applied because of its noise robustness and ability to reduce large numbers of trials into a single frequency response. 115 solo insect frequency domain identifications were conducted. A representative example is shown in camera and 3D views in Fig. 2a–c and in the Supplementary S1 Video. 3D position coordinates shown in Fig. 2b illustrate the *x* coordinate tracking behavior.

**Figure 3:**
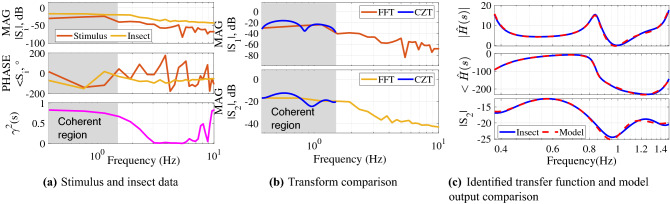
Frequency domain system identification example. (**a**) Magnitude, phase, and coherence $\gamma ^2$ plots of the input stimulus $S_1$ and output (insect) position $S_2$. A linear relationship between stimulus and insect position is indicated in the shaded region below 1.5 Hz where $\gamma ^2 > 0.6$, (**b**) Comparison of Fourier and Chirp Z-transform magnitudes for stimulus $S_1$ and insect $S_2$ in the region of highest coherence, (**c**) The identified transfer function $G_e(s)$ (dashed red) shows strong agreement with the measured frequency response $\hat{H}(s)$ (blue) in both magnitude and phase, as do true and identified model output $|S_2|$.

Frequency transformed stimulus and output signals $S_1$ and $S_2$ (respectively) are used to construct a frequency response function, shown in magnitude and phase components for both FFT and CZT transforms in Fig. 3a. A coherent region of response below 1.8Hz is visible, indicating that the input and output have a strong linear relationship (as quantified by $\gamma ^2>0.6$) in this range. Some deviation from ideal tracking (0dB magnitude, 0$^\circ$ phase) is visible in Fig. 3a, with gain showing some overshoot and a negative phase (lag) indicating a reaction time that quantifies the combined effect of airframe dynamic response due to physics and visuomotor delay. The CZT transform was used to improve resolution in the strongly coherent range (Fig. 3b) below 1.15Hz and the CZT-derived frequency response function $\hat{H}(s)$ used to identify the equivalent transfer function (refer to ﻿“Methods: System identification”).

The fit error statistics in Table 2 indicate the transfer function structure that best models the example trajectory; in this case, a 3 pole, 3 zero transfer function with 15 ms processing latency (transport delay) best models this example trajectory. After the VISIONS tracking algorithm records the 3D insect flight trajectories, the system identification method determines the dynamic model best describing those trajectories. Thus the transfer function characterization (3 pole and 3 zero in this example) is an identification result (rather than being a structure prescribed a priori or a tracking algorithm outcome). The identified model is

1 $$\begin{aligned} G_e(s) = e^{-.015s}\frac{0.7377s^3+0.5992s^2+1.175s+0.2213}{s^3+0.544s^2+1.85s+0.2594}, \end{aligned}$$

and a comparison of this identified model and experimental response functions in Fig. 3c show the strong agreement indicated by Table 2. Measured and frequency domain modeled outputs $S_2(\omega )$ show good agreement in Fig. 3c.

**Table 2:** Model candidates and performance for an example insect.

Model struct.	Fit	FPE	MSE	Delay
2 pole, 1 zero	33.47%	7.77$\times 10^{-3}$	7.09$\times 10^{-3}$	50ms
3 pole, 3 zero	95.06%	4.56$\times 10^{-5}$	3.90$\times 10^{-5}$	15ms
4 pole, 3 zero	42.21%	6.44$\times 10^{-3}$	5.31$\times 10^{-3}$	2ms

#### Example: Time domain identification

**Figure 4:**
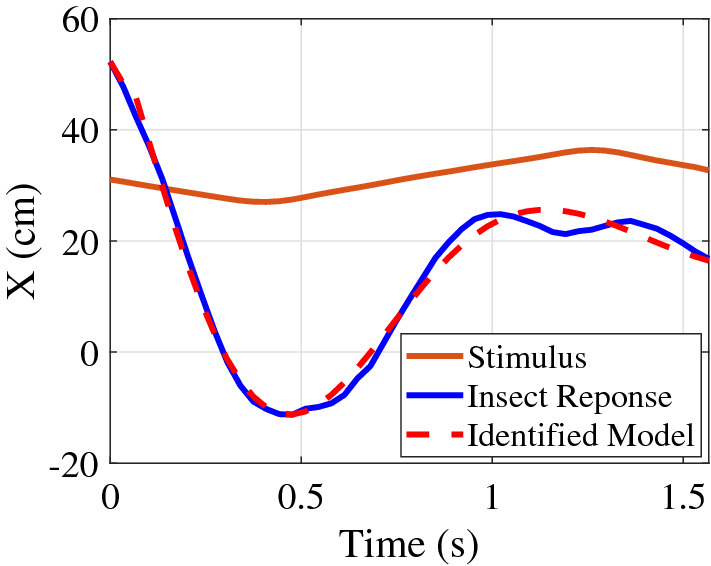
Example of time domain identifications used for trials $\le 1.5$ sec show tracking performance.

For short trajectories ($\le 1.5$ s), time domain system identification was performed (see “Methods: System identification”). Fig. 4 shows an example time domain system identification that identified a model

2 $$\begin{aligned} G_e(s) =e^{-.04s}\frac{-5.462s+39.42}{s^2+4.471s+75.86} \end{aligned}$$

with a fit percentage of $88.13\%$ and FPE of $1.12\times 10^{-3}$.

### Group dynamics identification

**Figure 5:**
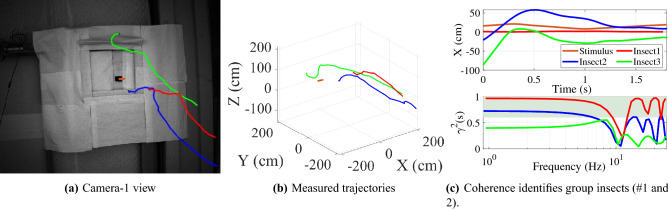
Segmentation of group insects by coherence. (**a**) Three insect flight trajectories in entrance approach as seen by camera-1, (**b**) Reconstructed 3D flight paths and stimulus, (**c**) Insect flight trajectories and coherence. Insect1 and Insect2 are identified as group insects due to coherence $\gamma ^2 > 0.6$ (shaded area). Insect3’s coherence is below 0.6 as it was not entering into the entrance and it was not considered as a group insect.

We also identified individual insect dynamics during group approaches to the entrance. An example of group behavior is shown in Fig. 5 and in the S2 video. In this example, insect-1 and insect-2 are identified as a group due to their high coherence ($\gamma > 0.6$ up to 5 Hz). The same frequency and time domain identification tools were applied to extract each individual insect’s dynamic model and corresponding time delay.

**Table 3:** Comparison of pure delay and linear approximation.

Delay model	Trials	Mean FIT	Standard deviation
Pure delay	77%	86.07	13.54
Linear approximation	23%	83.21	11.17

### Full dataset and delay distributions

**Figure 6:**
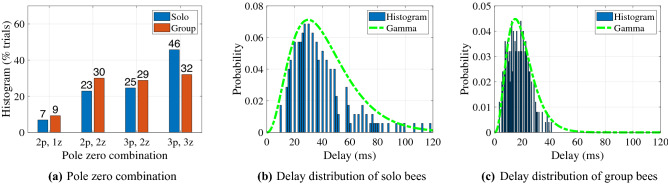
(**a**) Distribution of identified model structures, as indicated by number of poles and zeros, (**b**) histogram of solo insect delays vary from 0 to 120 ms and normalized gamma distribution, (**c**) histogram and fitted distribution curve in group bees, containing a narrower band of delays from 0 to 45 ms.

We used the stimulus and insect trajectories to find the delay by considering model structures that included a delay in both system identification paths. Individual delay identification was an outcome of Eq. (5) (for frequency domain) and Eq. (10) (for time domain). When the identification process was applied to the full dataset of 450 trials, some variation in identified model structure was seen, as in shown in Fig. 6a. As with the solitary insect example (Table 2), the identification proved insensitive to fit criteria choice (ie, $\max (\textrm{FIT}),\min (\textrm{MSE}),\min (\textrm{FPE})$) across insects measured in this study. When a transfer function model was fit to the frequency range of the experimental frequency responses having high coherence, best-fit model for all insects in terms of pure tracking delay and linear approximation was seen in Table. 3. The pure delay model outperformed the linear delay model and was used in the subsequent analysis.

The relative frequency of the identified delays shown in Fig. 6b,c show the discovery of two clear effects. First, the insects’ visuomotor tracking delays are heterogeneous, varying from 7ms to 120 ms. Second, insects operating in group settings show a different distribution of delays than those in solitary environments, in particular a significantly narrower distribution. The solitary delay distribution shows a mean of 30 ms and standard deviation of 50 ms, while the group delay distribution has an 18 ms mean with a significantly narrower 8ms standard deviation.

We also verified the robustness of the identification method against measurement noise in the recorded data. For this, we used simulated trajectories (see Supplementary Fig. 1a) generated by a similar known transfer function and added Gaussian noise of varying magnitude (refer to Supplementary Fig. 1b). The system identification performance for different signal to noise ratios (SNR) is shown in Supplementary Fig. 1c. The results show that the system identification technique fit quality exceeds 95% for signal-to-noise ratios above 10, an effect that is predicted by the theoretical noise robustness of frequency domain identification methods^54^. We also investigated the sensitivity of the results to process and measurement noise filter parameters. As seen in Supplementary Fig. 2, the filter parameters do not meaningfully affect the results.

The combination of both frequency and time domain system identification methods allowed us to both increase the size of datasets considered beyond those meeting the requirements of each approach, and to also verify that the results were insensitive to the choice of time or frequency domain. Strong system identification results require attention to provide input stimulus design that injects sufficiently strong signals diverse enough to excite the internal dynamics, motivating the novel experimental stimulus introduced in this work. The resulting insect flight trajectories and associated frequency components richness helped provide strong identification results.

## Discussion

This study quantified for the first time that the presence of neighbors is associated with in-flight synchronization in honeybee visual reaction time. Based on the theoretical analysis, an emerging **hypothesis (H2)** could be that *the insects respond to the presence of other agents by adjusting their reaction times to support cohesion*. Such a hypothesis requires a two-part test, that of neighbor awareness and establishing a connection to cohesive behavior. Numerous studies demonstrate that insects are influenced by group size and structure. Rooke 2020’s behavioral assays in walking *Drosophila* indicated that flies can sense both group size and density, and that olfaction may be important for determining group size (when compared to previous visuo-acoustic sensory studies)^55^. *Drosophila* show that behaviors including sleep habits, movement, social spacing, and pairwise interactions are affected by the presence of neighbors (largely by olfaction), with some authors labeling these outcomes as “peer pressure”^55–57^. Previous literature, while limited to walking and confined insects, thus supports the neighbor presence awareness component of hypothesis H2. The mechanism test is more involved, as observation of two coupled effects is not sufficient to establish causation. In the following section, we develop the necessary swarm theory to establish a plausible hypothesis for adjustment (refer to Method:  section “Swarm model”).

To understand the effect of these changing delay distributions in the swarm context, it is helpful to describe the measured distributions functionally. To provide sufficient generality to cover common distributions (e.g., Gaussian or Poisson), we used a Gamma distribution representation having shape parameters $m=3.5$, $a = 12$, $\tau _m = 1.85$ ms in the solo case and $m = 4$, $a = 5.1$, $\tau _m = 4.1$ ms in the group case, as illustrated in Fig. 6. For these distribution parameters, we applied mean field and parametric bifurcation analysis to find the swarm model’s stability regions (refer to Method: “Mean field analysis” and “Hopf bifurcation”). The result (Method Eqs. (32) and (33)) was a parametric description of the stability contour as a function of egocentric influence $\alpha$, neighbor influence $\beta$, and swarm center oscillation frequency $\omega$ as

$$\begin{aligned} \alpha (\omega ) =&-\frac{\omega }{\tan {(\omega \tau _m - m\tan ^{-1}(\omega /a))}}\,, \\ \beta (\omega ) =&-\frac{\omega }{\cos ^m{(\tan ^{-1}(\omega /a))} \sin {(\omega \tau _m + m \tan ^{-1}\frac{\omega }{a}})}. \end{aligned}$$

**Figure 7:**
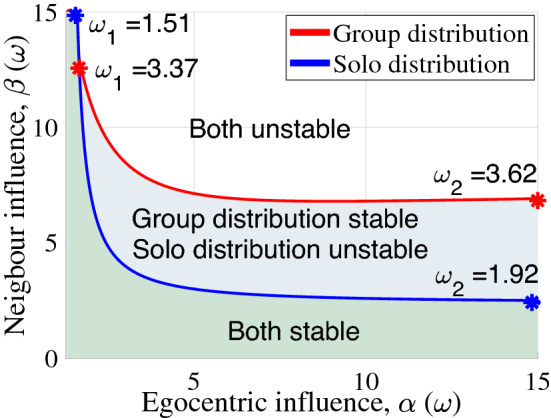
Stable and unstable regions for the two delay distributions (solo and group) were discovered by applying Eqs. (32) and (33) describing the stability boundary in terms of an individual agent’s ego influence $\alpha$ and neighbor influence $\beta$. Low neighbor influence factors lead to stable swarm center positions (green region) for either case. As neighbor influence grows, the swarm using a solo distribution loses stability (blue region) before the swarm using group behavior delays loses stability (white region), indicating that the group distribution provides a larger range of response weights (gains) that lead to a stable swarm. As the egocentric weight $\alpha$ grows on the stability boundary, the transition oscillation frequency $\omega$ rises.

The stability regions in Fig. 7 (additional views in Supplementary Fig. 3) show that for low values of both egocentric and neighbor influence $\alpha$ and $\beta$, the swarm remains stable regardless of delay distribution, and high values of neighbor influence are similarly destabilizing. However, the boundary of swarm stability when using the group delays (red curve) shows a higher tolerance for neighbor influence than the solitary delay distribution (blue curve). This finding suggests that one function of the delay distribution adjustment seen in group interacting insects is to support cohesion by improving the margin of destabilization. $\omega$ indicates the swarm center’s oscillation frequency at the stability boundary, and shows that the delay adjustment also affects the oscillation frequency. In both cases, oscillation frequency on the stability boundary increases with egocentric influence. Group delays show higher oscillation frequencies, indicating that group delays support a higher frequency motion before destabilizing. In many of the recorded experiments, individual trajectory oscillations were observable; current work quantifying the trajectories indicate they are below the limit frequencies in Fig. 7, again suggesting the insects remain below the stability boundary. This approach to assessing the impact of delays in swarms by weighing insect individuality against neighbor reaction strength incorporates the shape of delay distribution. While this portion of the theoretical outcome may be intuitive, previous theoretical models often do not consider heterogeneous delays, and rigorously showing the outcome holds for a relevant model did require developing a new approach, particularly to achieve the generality needed to account for gain variation (ie, to show that the result persists regardless of the insects’ strength of ego vs neighbor reaction). (The theoretical development scope resulted in the theoretical method spanning seven blocks in Fig. 10). Our study indicates that the solitary reaction time distribution leads to a less coherent swarm, providing some agreement with purely theoretical work^50^.

**Figure 8:**
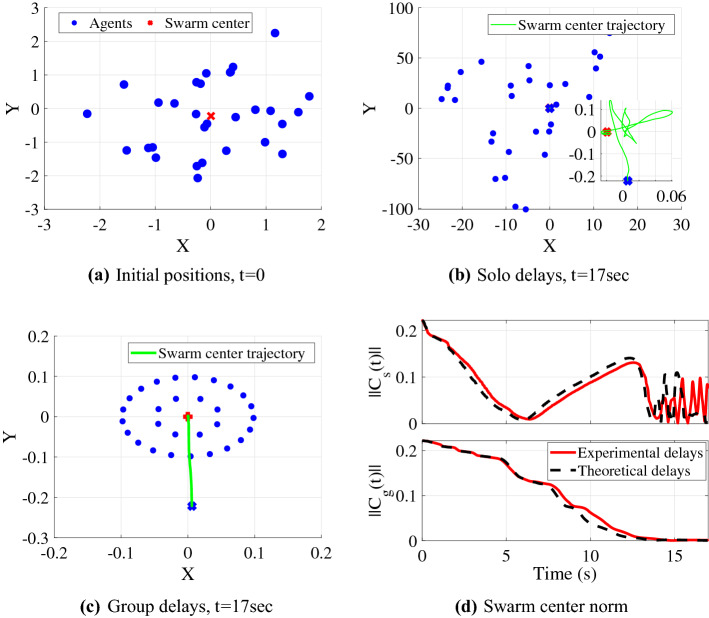
Swarm simulations with experimental delays for solo and group behaviors. (**a**) Random initial positions of 30 agents, (**b**) ending position of agents using experimental solo delays, (**c**) ending position of agents using experimental group delays (**d**) time histories of swarm center $C_s(t)$ and $C_g(t)$ norms for solo and group delays, respectively.

Some approximation could be introduced by the swarm model and the gamma distribution fit process, and while the theoretical analysis can assess barycenter stability, it cannot yet predict relative positions or formation. To explore the validity of the swarm model and examine the effects of the experimental delays, we simulated an interconnected visual swarm with both experimentally-quantified delays and those drawn from the gamma distribution fits, each of which began from randomly distributed initial positions in Fig. 8a. Simulations using the heterogeneous delays measured in both solo and group delays (ie, the measurements in Fig. 6) are shown in Fig. 8 for 100 agents. For theoretical delays the solo swarm used the gamma distribution fit parameters as $\alpha = 7$, $\beta = 6$ and $\frac{m}{a} = .3$, while group used the same $\alpha ,\beta$ and $\frac{m}{a} = 0.78$. The repulsive potential’s amplitude and applied distance were taken as $B_r = 0.5$ and $D_r = 1$. The time history of swarm center position in Fig. 8d shows that the swarm center stability failure seen in theoretical analysis is replicated for simulations using the discovered delays. Additionally, the simulations show that the instability extends to formation, with group-measured delays stabilizing to a formation in Fig. 8c and solo-measured delays leading to a motion having no observable formation in Fig. 8b.

While a rigorous theoretical analysis shows that the measured adjustment stabilizes a swarm position barycenter, there are other reasons that could underlie the adjustment, such as peer pressure, collision avoidance, or inter-agent competition. We observed in our dataset that the presence of neighbors reduced the amplitude of in-flight lateral oscillations relative to solo flyers, suggesting that collision avoidance may be a consideration^55,58^. Conversely, in-flight midge interactions showed effective forces were attractive^23^, lending support for the choice of an attraction (repulsion) swarm model used in this study.

An **related hypothesis (H2a)** could be that *the insects possess an internal clock (oscillator) and in flight social communication (such as implicit visual communication) regulates their behavior to that of the group*. Hypothesis H2a again requires a two part test. The presence of oscillators in insects is well-established, including discrete (binary) oscillators in firefly light outputs^59^. The finding of “selective attention” mechanisms that provide phase locking of central complex to flickering visual stimuli suggests that the visual stimuli provided by other agents, including those tracking the hive entrance stimulus in this experiment, could serve to provide a reference signal for synchronization^60^. Finally, an **alternate hypothesis (H3)** may be constructed that posits no insect adjustment is occurring (i.e., their behavior is maintained), and that the presence of other neighbors tracking the stimulus provides a richer visual stimulus. H3 could be stated as *the insect’s core tracking is maintained, and a richer input stimulus of neighbor motion improves individual tracking latency.* While contemporary work suggests that the presence of neighbors may modify individual performance without direct adaptation, these studies have generally focused on how hydrodynamic interactions enable metabolic^61^ or formation^62,63^ phenomena. A theoretical and experimental basis to verify an analogous effect on tracking performance via information interactions in flying insect groups is comparatively less mature.

**Contribution of this paper** Previous work quantifying three-dimensional position, velocity, and acceleration of group flight has documented the complexity of coordination, including low polarization and correlation levels^64^, and significant work remains to provide theoretical interpretation of these observations. Larger scale measurements including external group stimulus analyzed these at the macro (swarm-level) scale rather than developing individual dynamics models at the agent scale. Examples of macro-scale experiments include those using acoustic stimulus to support linear and spectral analysis^65^ and visual stimulus to analyze the group’s effective material properties^21,66^. A step forward was found in Kasper 2019^21^, which creates a parallel simulation from stochastic differential equations, although the study does not yet analyze the experimental data at the individual scale or provide a theoretical interpretation. Previous system identification of insect individual visual processing delays reported population average delays, connecting them to environmental change (luminance level) rather than analyzing how the delays affect inter-agent interactions or flight performance^25,67^.

This study discovers a new neighbor-induced reaction time adjustment, testing hypothesis H1, and develops theory to demonstrate its value to swarm coordination as a plausible explanation and function. The study is the first to analyze flying insect digitized group trajectories at the individual scale by coordinating experimental system identification, theoretical development and computational simulation to yield the most comprehensive picture to date of how delays are modified in group flight and their role in coordination. Conversely, current knowledge retains some ambiguity in the mechanism underlying the experimental effect. Having established this new effect and the plausibility of hypotheses (H2 and its alternate H3), this study places differentiating between mechanistic hypotheses within experimental reach.

## Summary

In summary, this study measured flying insects tracking a moving stimulus in solo and group behaviors and quantified the visuomotor delay (reaction time) in their closed loop tracking. We used a real-time camera-based tracking system that quantified both moving target and insect position in three dimensions. System identification tools were applied to 450 recordings to identify the closed loop tracking dynamics between stimulus motion and insect body motion, separating the effects of open loop plant (locomotion) physics from visuomotor feedback delay and quantify the visuomotor delay as a transport delay. The measured insect sensorimotor delays were used to find a delay distribution across population, showing that insect visual sensorimotor feedback delays in a tracking task are heterogeneous across population, consistent with indoor experiments^53^, and disproving hypothesis H1 that an indoor/outdoor environmental change would impact the solitary distribution. Instead, significantly more variation (50ms standard deviation) was found when an insect was the only animal tracking the target, relative to group behaviors in which multiple insects tracked the target (8ms standard deviation). To develop a hypothesis (H2) incorporating the implications of the measured delays on visually-guided swarms, we then integrated the measured delays into a visually interacting swarm model. Analysis on this model indicates conditions needed for the center of mass’s position and allows us to map the stable and unstable regions as a function of behavior. Simulations were conducted using theoretical fits to the delays (gamma distribution) and the experimental delays. The analysis and simulation indicate that the processing delays measured in solitary conditions yield an unstable swarm behavior and that the group delays provide a stable center of mass and cluster shape, and predict the speed of swarm center oscillations at transition. An alternate hypotheses (H3) regarding an increase in visual information has mild literature support.

Overall, this study quantifies response delays in solo and group tasks and connects these measurements to theoretical limits on the allowable delays for insects in visually guided swarms. The results of the delay identification suggest the insects operating in group contexts could adjust their delays to support swarm cohesion (H2). The finding that delay is reduced in group flight also raises questions about how implicit communication transfer in groups can improve individual performance (H3). This consistency between experimental measurements of solo and group tasks in flying insects with theoretical and simulated frameworks quantifying constraints is an important outcome for understanding how flying insects support swarming motions despite tight constraints on sensing and feedback. The results provide a foundation for swarming aerial robotics with limited computational resources. For these systems, in which processing delays are significant, knowledge of what delay distributions support stable motions will guide engineers in distributing processing tasks appropriately.

## Methods

Figure 10 depicts the overall study, including experimental work, theoretical analysis, and simulations. The stimulus is a horizontally moving beehive entrance as shown in Fig. 1a. The measurement system consists of three cameras with angular separations from 50 to 90 degrees as seen in Fig. 1b. Honeybees were recorded entering and exiting the hive entrance as in Fig. 1d. Insects were recorded while approaching the entrance in both solitary and multi-agent conditions. The experimental apparatus consists of a moving entrance stimulus design and an imaging system.

**Figure 9:**
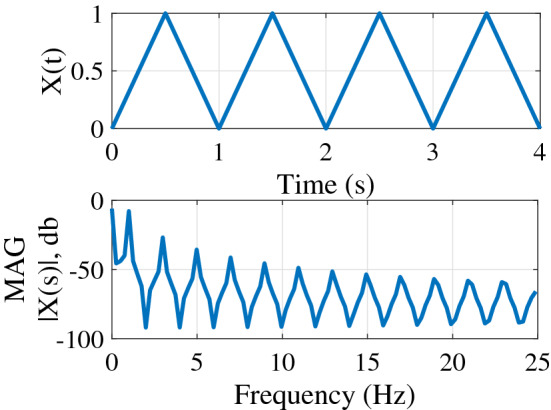
A triangular signal was used to apply horizontal entrance movement along the X axis of the world frame, resulting in rich frequency components.

**Figure 10:**
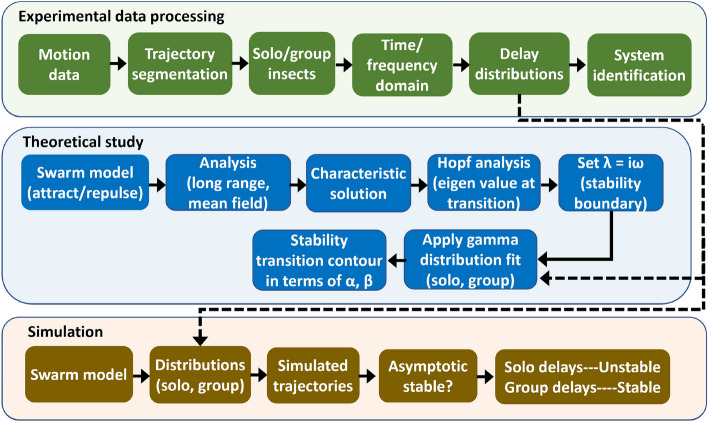
Overall study incorporates three components: a multi agent tracking experiment, theoretical analysis, and simulated performance.

### Stimulus design

The entrance stimulus was a 2-inch square hole (tunnel entrance) that oscillates horizontally along the world frame’s *X* axis. This setup included two stepper motors (Nema 23) and a motor driver (a4988), which was controlled by a micro-controller (Arduino Uno). The circuit diagram is depicted in Fig. 1c which directs the motors according to triangular frequencies as seen in Fig. 9. Previous confined indoor experiments used a sum of sinusoids stimulus movement to identify solitary tracking dynamics^53^. In preliminary work, we tested a sum of sinusoids signal that provided results similar to those reported in this study, but restricted the number of datapoints as hive tracking behaviors in outdoor conditions were shorter. The lack of confinement and clear behavioral exit strategy (entering the hive) limits the tracking duration. Triangular waves are a sum of multiple sine waves restricted to only odd harmonics, and taking advantage of the shorter rise time of the triangular waves was helpful to provide good frequency content in this limited time. The inflection point in the triangular wave motion contains the frequency content diversity and signal strength considerations then required limiting our analysis to recordings that included the reversal.

### Imaging system

**Figure 11:**
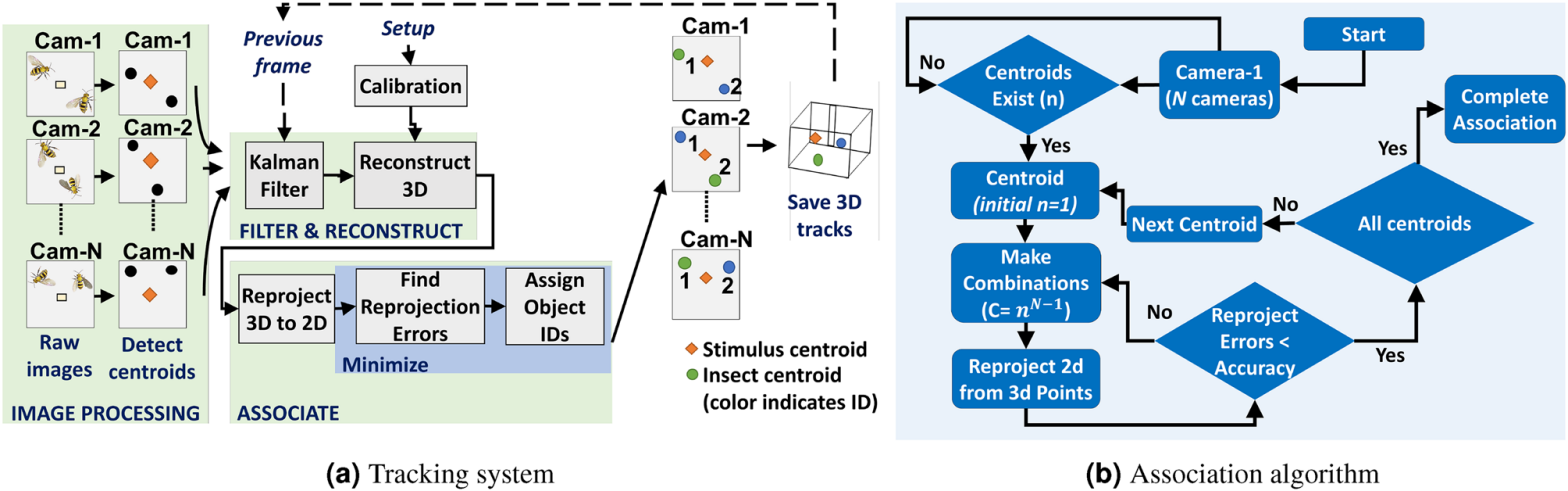
Flowchart of tracking (**a**) and association algorithms (**b**) for VISIONS multi-agent tracker.

The VISIONS^53^ tracking system was used to record insect trajectories during solitary and group flight conditions. This study updates the tracker to track multiple insects. The functions in the tracking system are shown in Fig. 11a. To expand tracking from prior single agent tracking with VISIONS to multiple insects, we incorporated data association. The functions in the association algorithm are shown in Fig. 11b. Visual occlusions or imperfect image segmentation may transiently impact visually tracked data, and a mechanism to recover during transient data dropouts is helpful. As in Islam 2022^53^, VISIONS applies a Kalman filter during transient dropouts. To ensure the Kalman filter’s Gaussian model of process and measurement did not affect the identification results, the robustness of the results to filter parameter variation was also verified.

The intrinsic and extrinsic parameters of the cameras were calibrated via bundle adjustment^68^. For each insect, we calculate reconstructed 3D points from all possible combinations of 2D points and then reproject them from 3D to 2D. We use the intended match from this error list if the re-projection error (difference between observed and re-projected 2D points) is less than the desired accuracy. A set of *m* insects’ 2D positions of camera-1 at time *t* is described as $H_{1,t} = \{h^1_1(t),h^2_1(t),h^3_1(t),...h^m_1(t)\}$, where $h_1^{1...m}(t)$ represents their positions. Between camera-2 and camera-$N_c$, the possible combinations for each insect in $H_{1,t}$ is $c = m^{N_c-1}$. Here, $N_c$ is the total number of cameras. The re-projected 2D points vector is $D_{1..j} = \{d_1, d_2, d_3, ....., d_j\}$, where $d_j$ is the re-projected 2D point for $j = 1,...,c$. The associated agent is taken to be

3 $$\begin{aligned} {{\,\textrm{associate}\,}}{\left( h_1^m(t)\right) } = \min _{\begin{array}{c} j \end{array}=1,2....c} \Vert h^m_{1}(t)-D_{1..j}\Vert < \sigma \ \,, \end{aligned}$$

where $\sigma$ is the desired accuracy and $\Vert .\Vert$ is the 2 norm (Euclidean distance).

### System identification

We identify systems in both the time and frequency domains between the movement stimulus and the insect position. Frequency domain identification is accomplished when record lengths are sufficient (>1.5sec) and time domain when records are shorter.

#### Frequency domain identification

**Figure 12:**
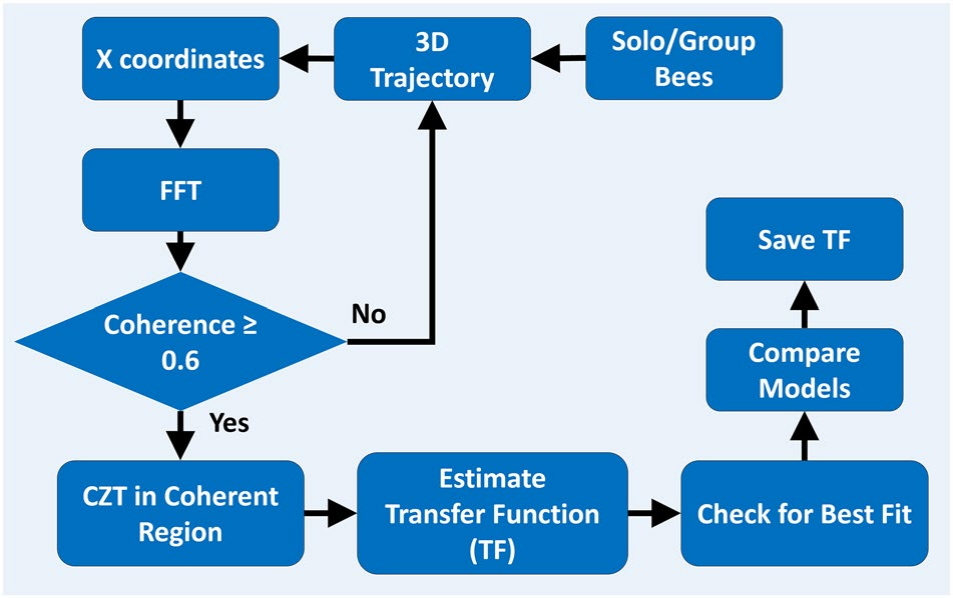
Frequency-domain system identification flowchart.

When the trajectory had sufficient frequency content (trial length), we applied the identification approach for the transfer function *G*(*s*) of the insect’s position response to stimulus motion via Chirp Z transform (CZT) developed in our prior work^53^ and illustrated in Fig. 12.

In the frequency domain, frequency response of target tracking is described by gain, phase, and coherence. Coherence $\gamma ^2(s)$ is calculated from the spectral and cross-spectral densities of input and output signals as

4 $$\begin{aligned} \gamma ^2(s) = \frac{|P_{xy}(s)|^2}{ P_x(s)P_y(s)}, \end{aligned}$$

where $|P_{xy}(s)|$ denotes the magnitude of the cross spectral power density and $P_x(s)$ and $P_y(s)$ represent the auto power spectral density of the stimulus (input) and bee (output) coordinates, respectively. The coherence between the stimulus and insect trajectories was used to determine the linear connection throughout the frequency range. We use the frequency response function obtained from the CZT transform by reference to a transfer function with an internal delay time $\tau$ to examine the flight dynamics and visuomotor delay of the tracking behavior. Frequency domain analysis prescribes a minimum record length $T_{rec}$ as a function of target identification frequency; $T_{rec} > \frac{2\pi }{\omega _{min}}$ is normally sufficient to resolve a model having minimum frequency $\omega _{min}$^54^.

We conduct the system identification technique across several possible estimated transfer functions $G_e(s)$ and varying time delays $\tau _i \in [0, 200]$ ms. A processing delay $\tau _i$ could be modeled as a pure tracking delays $(e^{\tau _is})$ or linear approximation $\left( \frac{1}{1+\tau _i s}\right)$ in the transfer function. We included both delay structures in the system identification framework to compare the results. The identified transfer function model is found from the minimum absolute difference between true and model transfer functions over region of coherence, which is presented as

5 $$\begin{aligned} \min _{\tau _i,G_e} |\hat{H}(s) - G_{e}(s)M(s,\tau _i)|_{s=j\omega ,\;\omega = \text {arg}\{\gamma ^2(s) > 0.6\}}, \end{aligned}$$

with delay model structure $M(s,\tau _i) = \{e^{-s\tau _i}, \frac{1}{1+\tau _is}\}$. Here, $\hat{H}(s)$ represents the measured frequency domain data that was derived by dividing the frequency domain versions of the bee and stimulus trajectories, denoted by $\hat{H}(s) = \frac{D_2(s)}{D_1(s)}$. Three fit criteria FIT, MSE (mean square error) and FPE (final prediction error) are used to determine the best dynamics model^53^.

#### Time domain identification

For the time domain system identification, normalized least square estimation is used to find the dynamic system^69^. The discrete time domain representation of the stimulus and insect are $x_1(k)$ and $x_2(k)$, and by considering a unknown transfer function it can be written as

6 $$\begin{aligned} \frac{x_2(z)}{x_1(z)} = \frac{b_1z^{-1} + b_2z^{-2}+...b_nz^{-n}}{1+a_1z^{-1}+....+a_nz^{-n}}, \end{aligned}$$

where the unknown coefficients are $a_i, b_i; i=1...n$. The time domain solution can be written as

7 $$\begin{aligned} \begin{aligned} x_2(k)& = -a_1x_2(k-1)....-a_nx_2(k-n)\\ &\quad + \, b_1x_1(k-1)+...+b_nx_1(k-n). \end{aligned} \end{aligned}$$

Then we construct a parametric model, $x_2(k) = \phi ^T(k)\theta ^{*}$ where

8 $$\begin{aligned} \theta ^{*} = [a_1 \, a_2 \,.....\,a_n \, \, \, \,b_1 \, b_2.......\,b_n]^T, \end{aligned}$$

9 $$\begin{aligned} \begin{aligned} \phi (k) = [-x_2(k-1)\,....\,-x_2(k-n) \\x_1(k-1)\,....\,x_1(k-n)]^T. \end{aligned} \end{aligned}$$

The cost function is written as

10 $$\begin{aligned} J = \sum _{k=0}^{M-1}(x_2(k)-\phi (k)^T\theta ), \end{aligned}$$

where $k=0....M$. The solution $\hat{\theta }=\mathop {\textrm{argmin}}\limits _\theta {J(\theta )}$ is

11 $$\begin{aligned} \hat{\theta } = \sum _{k=0}^{M-1}(\phi (k)\phi (k)^T)^{-1}\sum _{k=0}^{M-1}\phi (k)x_2(k). \end{aligned}$$

The fit criterion is defined as

12 $$\begin{aligned} f = 100 \times \frac{|x_2-\hat{x_2}|}{|x_2 - \mathrm {mean(x_2)}|} \end{aligned}$$

where $\hat{x_2}$ is the predicted output.

### Robustness of identification strategy to noise and filter parameters

We conducted two tests to verify the robustness properties of the identification method. First, noisy data was produced by adding Gaussian noise to simulated trajectories. We generated an example simulated trajectory by three poles and three zeros transfer function with an arbitrary input signal, and added white Gaussian noise to the signal. For an input signal *U*(*t*) and simulated output *Y*(*t*), the noisy output was generated via $Y_n(t) = Y(t) + w(t)$, where *w*(*t*) is the additive white Gaussian noise. The signal-to-noise ratio |*Y*|/|*w*|, abbreviated SNR, was used to quantify the noise size.

Secondly, the Kalman filter’s process and noise design parameters were adjusted to verify they did not affect the results. Measurement noise and process noise covariances in the Kalman filter can be found from the standard deviation of the position $\epsilon =(\epsilon _x, \epsilon _y$) and acceleration ($\epsilon _a$)^53^ . The primary result showed in Fig. 6 used $\epsilon$= 0.2 and $\epsilon _a = 0.8$. Identification robustness to assumed noise levels was tested by increasing $\epsilon$ while holding $\epsilon _a$ constant.

### Swarm model

We can construct a visually interconnected swarm with agents experiencing visual processing delays by applying a first order dynamic system model with *N* number of agents and postulating that the agents experience delayed interconnections with each. We build upon previous models with constant delays^50^ to develop a swarm model with heterogeneous delays and weighting factors. The swarm model is

13 $$\begin{aligned} \frac{dx_i}{dt}(t) =&-\nabla _iA^a(x_{ij},\tau _{ij})- \nabla _iA^r(x_{ij},B_r,D_r)\,, \end{aligned}$$

where $x_i$ is (vector) position of the agent *i*, $\nabla _iA^a(x_{ij},\tau _{ij})$ and $\nabla _iA^r(x_{ij}, B_r, D_r)$ are attractive and repulsive potentials respectively, specified as

14 $$\begin{aligned} A^a (x_{ij},\tau _{ij}) = \frac{1}{2}\left( \sum _{i=1, i\ne j}^{N}(\alpha x_i(t)-\beta x_j(t-\tau _{ij}))\right) ^2 \end{aligned}$$

and

15 $$\begin{aligned} A^r(x_{ij},B_r, D_r)&= \sum _{i=1, i\ne j}^{N} B_re^{\frac{-1}{D_r} {\Vert {\alpha x_i(t)-\beta x_j(t-\tau _{ij})}\Vert }} . \end{aligned}$$

Here, $\tau _{ij}$ is the delay from agent *i* to agent *j*, $\alpha$ is the egocentric influence weight and $\beta$ is the neighbor influence weight. $B_r$ and $D_r$ are the amplitude and applied distance of repulsive potential. At long range, stability may be determined by only the attractive potential and conversely at short range, stability may be determined by considering only the repulsive potential^41,53^. So, for the mean field analysis we consider only the attraction potential. The center of mass of the swarm can be defined as a vector *C*(*t*) as

16 $$\begin{aligned} C(t) = \frac{1}{N} \sum _{i=1}^{N}x_i(t) . \end{aligned}$$

To find the position stability of the swarm, the norm of swarm center is found as $\Vert C(t)\Vert = \sqrt{C^2_{x}(t)+C^2_{y}(t)}$, where $C_{x}, C_{y}$ are the *X* and *Y* coordinates of the swarm center respectively. Swarm center stability is then $\Vert C(t)\Vert \rightarrow \textrm{constant}$ as time $t \rightarrow \infty$.

### Mean field analysis

Each agent is updated as

17 $$\begin{aligned} \dot{x_i}(t) = -\frac{1}{N}\sum _{j=1, i\ne j}^{N} ( \alpha x_i(t) - \beta x_j(t-\tau _{ij})). \end{aligned}$$

The position of each agent $x_i(t) = C(t) + \delta x_i(t)$ and $\delta x_i$ is the deviation from the center of mass *C*(*t*). The position derivative $\dot{x_i}$ is written as

18 $$\begin{aligned} \begin{aligned} \dot{x_i}(t)&= \dot{C}(t) + \delta \dot{x_i}(t)\\&= -\frac{\alpha (N-1)}{N}[C(t)+\delta x_i(t)]\\& \quad +\frac{\beta }{N}\sum _{j=1}^{N} C(t-\tau _{ij})+\delta x_j(t-\tau _{ij})].\end{aligned} \end{aligned}$$

Summing over *i* and taking $\sum _{i}^{N} \delta x_i(t) = 0$,

19 $$\begin{aligned} \begin{aligned} \dot{C}(t)&= -\frac{\alpha (N-1)}{N}[C(t)+\delta x_i(t)]\\& \quad +\frac{\beta }{N^2}\sum _{i=1}^{N}\sum _{j=1}^{N} C(t-\tau _{ij})+\delta x_j(t-\tau _{ij})]. \end{aligned} \end{aligned}$$

We can obtain the center of mass by approximation considering a double sum^51^. The discrete terms can be approximated by a distributed density function $g_{\tau }(\tau )$. We can extend the discrete delays towards a distributed density function such as

20 $$\begin{aligned}{}&\frac{\beta }{N^2} \sum _{i}^{N}\sum _{j}^{N} \delta x_{j}(t- \tau _{ij}) \\&= \frac{\beta }{N^2}(N-1)\int _{0}^{\infty } \sum _{j}^{N}\delta x_i({t-\tau })g_{\tau }(\tau )d\tau =0, \end{aligned}$$

with the same approximation made for the center of mass *C*(*t*). For large number of agents, $\frac{N-1}{N} \approx 1$ (0.97 in this study’s simulations). Finally, we can write the swarm center dynamics as

21 $$\begin{aligned} \dot{C}(t) = - \alpha C(t) + \beta \int _{0}^{\infty } C(t-\tau )g_{\tau } d\tau , \end{aligned}$$

which is now a general differential equation in the form

22 $$\begin{aligned} \frac{d(C(t))}{dt} = F(C(t), \bar{C}(t)), \end{aligned}$$

where $\bar{C}(t)$ is a delay weighted state given by

23 $$\begin{aligned} \bar{C}(t) = \int _{\tau _m}^{\infty } C(t-\tau )g_{\tau } d\tau \equiv \int _{-\infty }^{t-\tau _m} C(t)g_\tau (t-\tau ) d\tau . \end{aligned}$$

Here, $\tau _m$ is the minimal delay and $g_\tau$ is the probability density function of the distribution, such that $\int _{0}^{\infty } g_\tau d\tau =1$.

### Hopf bifurcation

We want to examine the sensitivity of the swarm’s local stability to changes in distribution shape. The Gamma distribution was chosen because of its generality. One can find an equivalent Gamma distribution that represents many of the common distributions, such as Gaussian, Poisson, and is closely related to exponential, Erlang, Maxwell-Boltzman, and Wishart distributions. Thus, choosing the gamma distribution framework avoided the need to unfairly constrain the results to a more specialized distribution. The Gamma distribution has a relation between its mean with the shape. To examine local stability we need to linearize the system at a steady state solution. By taking $C(t) = ce^{\lambda t}$ in Eq. (21) we obtain the characteristic solution

24 $$\begin{aligned} \nabla {\lambda } =&\lambda + \alpha - \beta \int _{0}^{\infty } e^{-\lambda \tau _m} g_\tau (\tau ) d\tau = 0. \end{aligned}$$

We want to examine how delay affects stability, and apply the fact that the stable/unstable transition takes place when the characteristic equation has a root with zero real part. The density of the gamma distribution is

25 $$\begin{aligned} g_\tau (\tau ) = \left\{ \begin{array}{ll} 0, &{}0 \le \tau < \tau _m\\ \frac{a^m}{(m-1)!} (\tau - \tau _m)^{m-1}e^{-a(\tau -\tau _m)}, &{}\tau _m \le \tau \end{array}\right. . \end{aligned}$$

The unshifted density of the distribution can be written as $E= \int _{0}^{\infty } \tau g_\tau d\tau = \frac{m}{a}$, where parameters (*a*, *m*) specify the shape of the gamma distribution. The gamma distribution’s variance is $V = \frac{m}{a^2}$, and its Laplace transform is $G_\tau (\lambda ) := \int _{0}^{\infty } e^{-\lambda \tau }g_\tau (\tau ) d\tau = \frac{a^m}{(a+\lambda )^m}.$ Equation (24) may then be expressed as

26 $$\begin{aligned} \lambda + \alpha - \beta e^{-\lambda \tau _m}\frac{a^m}{(a+\lambda )^m} = 0. \end{aligned}$$

Linear systems lose stability when the roots of the characteristic equation cross the imaginary axis from left to right. To study the Hopf bifurcation, we take $\lambda = i\omega$ and $\tan {\theta } = \frac{\omega }{a}$ to get

27 $$\begin{aligned} i\omega + \alpha - \beta (\cos {\omega \tau _m} - i \sin {\omega \tau _m})\frac{a^m}{(a+i\omega )^m} =&0, \end{aligned}$$

28 $$\begin{aligned} i\omega + \alpha - \beta (\cos {\omega \tau _m} - i \sin {\omega \tau _m})\frac{1}{(1+i\tan {\theta })^m} =&0, \end{aligned}$$

29 $$\begin{aligned} (i\omega + \alpha )(1+i\tan {\theta })^m - \beta (\cos {\omega \tau _m} - i \sin {\omega \tau _m}) =&0. \end{aligned}$$

Applying de Moivre’s theorem^70^ and splitting the real and imaginary parts we obtain

30 $$\begin{aligned} \alpha (\omega ) =&\omega \tan {(m\theta )} + \beta \frac{\cos {(\theta )^m}}{\cos {(m\theta )}}\cos {\omega \tau _m}\,, \end{aligned}$$

31 $$\begin{aligned} \beta (\omega ) =&\frac{\omega + \alpha \tan {m\theta }}{\frac{\cos {(\theta )^m}}{\cos {(m\theta )}}\sin {\omega \tau _m}}. \end{aligned}$$

Finally, coupling the above two equations we may describe the stability contour as a function of egocentric influence $\alpha$ and neighbor influence $\beta$ as

32 $$\begin{aligned} \alpha (\omega ) =&-\frac{\omega }{\tan {(\omega \tau _m - m\tan ^{-1}(\omega /a))}}\,, \end{aligned}$$

33 $$\begin{aligned} \beta (\omega ) =&-\frac{\omega }{\cos ^m{(\tan ^{-1}(\omega /a))} \sin {(\omega \tau _m + m \tan ^{-1}\frac{\omega }{a}})}. \end{aligned}$$

Equations (32) and (33) allow one to map the stability transition contour for a given distribution (e.g., specified *E* and $\tau _m$ parameters), as a function of egocentric weight $\alpha$ and neighbour influence weight $\beta$ curves as a function of frequency $\omega$.

## Supplementary Information

Supplementary Video 1.

Supplementary Video 2.

Supplementary Figures.

## Data Availability

The data used in this experiment is available at https://figshare.com/s/d507bda7549cfd32f5d2.

## Nomenclature

$H_{i,t}$: Insects’ positions in camera-i
$N_c$: Number of measurement cameras
$D_{1..j}$: Re-projected 2D points
$h_{i}^m(t)$: 2D coordinates of insect m in camera-i
$P_{xy}(s)$: Cross power spectral density of signal *x* and *y*
$P_x(s)$: Power spectral density of signal *x*
$$\gamma ^2(s)$$: Coherence signal
$\hat{H}(s)$: Measured frequency domain data
$G_e(s)$: Estimated transfer function
$\tau _{i}$: Measured time delay of agent *i*
$$S_{1}$$: Stimulus trajectory
$$S_{2}$$: Insect trajectory
MSE: Mean square error
FPE: Final prediction error
$$\phi$$: Regression matrix
$$\theta ^{*}$$: Estimated parameter
$J(\theta )$: Cost function of time domain estimation
$f$: FIT error criterion
$D_r$: Applied distance of repulsive potential
$B_r$: Amplitude of the repulsive potential
$$A_r(x_{ij},B_r,D_r)$$: Repulsive potential
$$A_a(x_{ij},\tau _{ij})$$: Attractive potential
$N$: Number of agents
$\alpha$: Egocentric influence weight
$\beta$: Neighbor influence weight
$\tau _{ij}$: Delay from agent *i* to agent *j*
$C(t)$: Center of the swarm
$g_{\tau }$: Probability density function of distribution
$G_e(s)$: Estimated transfer function
SNR: Signal to noise ratio
$\epsilon$: Standard deviation of measurement noise in Kalman filter
