# Supplementary material for: Insect visuomotor delay adjustments in group flight support swarm cohesion
Source: Sci Rep. 2023 Apr 19;13:6407. doi: 10.1038/s41598-023-32675-5 (PMC10115836; doi:10.1038/s41598-023-32675-5)
Supplement: Supplementary file 3 — Supplementary Figures. [file 41598_2023_32675_MOESM3_ESM.pdf]

Supplementary Information

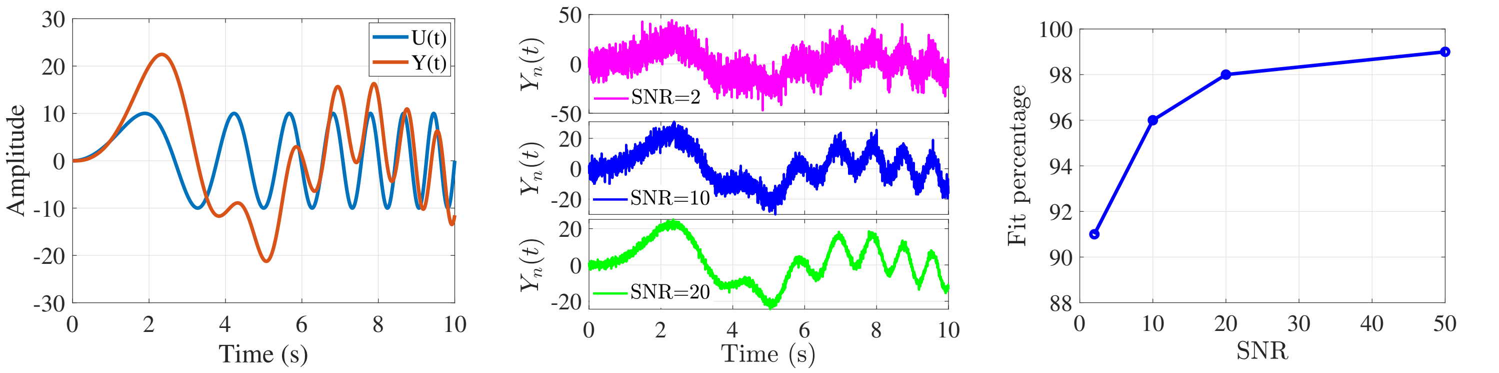

(a) Simulated example input and output

(b) Noisy output $Y_n(t)$ with variable SNR

(c) Fit percentage of identifying transfer function for different SNR

Figure 1: System identification of noisy simulated data.

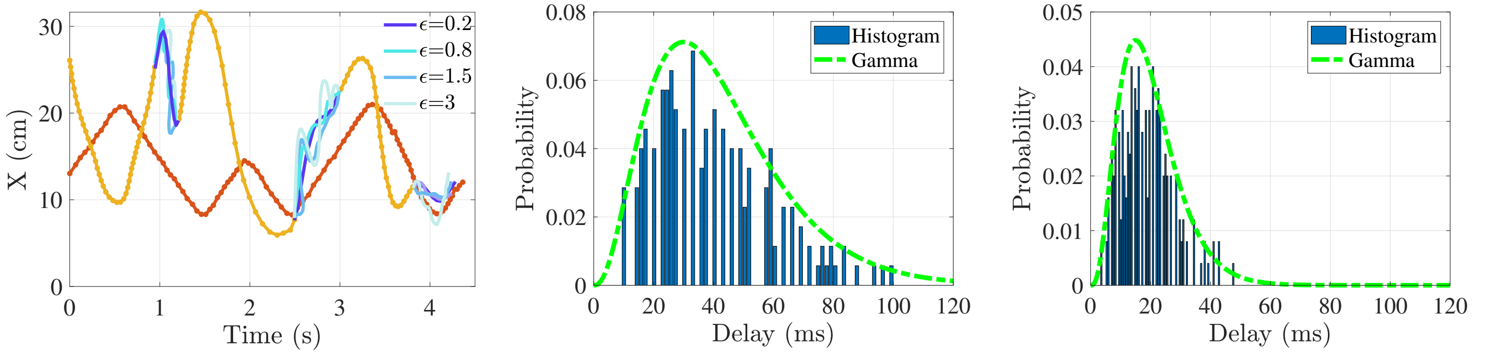

(a) X coordinate of an example insect (yellow), stimulus (orange), and Kalman filtered points for different standard deviation ϵ

(b) Delay distribution of solo bees at $\epsilon = 3$

(c) Delay distribution of group bees at $\epsilon = 3$

Figure 2: Sensitivity of noise filter on delay results.

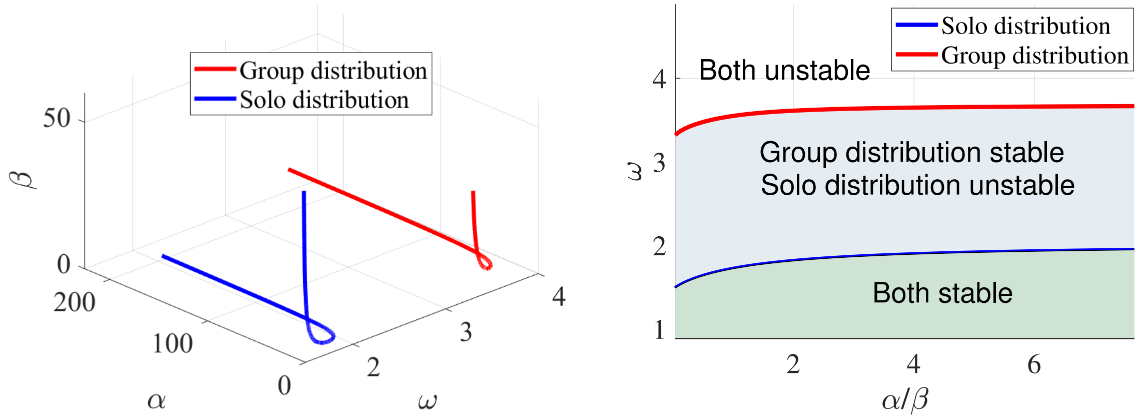

(a) 3D plot showing α, β as a function of changing ω

(b) Ratio of two weights

Figure 3: Additional views of stability regions.
